# Challenges and solutions to returning to clinical training after research: a multidisciplinary survey of integrated academic trainees in West Yorkshire, United Kingdom

**DOI:** 10.1186/s12909-021-02556-4

**Published:** 2021-02-18

**Authors:** C. L. Downey, J. Bentley, H. Pandit

**Affiliations:** 1 Leeds Institute of Medical Research at St James’s, St. James’s University Hospital, University of Leeds, Level 7, Clinical Sciences Building, Leeds, LS9 7TF UK; 2 School of Medicine, University of Leeds, Leeds, LS2 9JT UK; 3 Leeds Institute of Rheumatic and Musculoskeletal Medicine, University of Leeds, Chapel Allerton Hospital, Leeds, LS7 4SA UK

**Keywords:** Academic, Return to training, Support, Training

## Abstract

**Background:**

Time out of clinical training can impact medical trainees’ skills, competence and confidence. Periods of Out of Programme for Research (OOPR) are often much longer than other approved mechanisms for time of out training. The aim of this survey study was to explore the challenges of returning to clinical training following OOPR, and determine potential solutions.

**Methods:**

All current integrated academic training (IAT) doctors at the University of Leeds (United Kingdom) and previous IAT trainees undertaking OOPR in the local region (West Yorkshire, United Kingdom)(*n* = 53) were invited to complete a multidisciplinary survey.

**Results:**

The survey was completed by 33 participants (62% response rate). The most relevant challenges identified were completing the thesis whilst transitioning back to clinical work, the rapid transition between full-time research and clinical practice, a diminished confidence in clinical abilities and isolation from colleagues. Potential solutions included dedicated funds allocated for the renewal of lapsed skills, adequate notice of the clinical rotation to which trainees return, informing clinical supervisors about the OOPR trainee returning to practice and a mandatory return to standard clinical days.

**Conclusions:**

Addressing these issues has the potential to improve the trainee experience and encourage future trainees to take time out of training for research activities.

**Supplementary Information:**

The online version contains supplementary material available at 10.1186/s12909-021-02556-4.

## Background

There are more than 50,000 doctors undertaking postgraduate training in England [1]. At any one time, 10% of these doctors are taking approved time out of their training programme; this can be for a number of reasons including parental leave, sickness or bereavement, to gain additional experience or training outside of the postgraduate training programme, to conduct academic research, or to take a career break [1].

It has been suggested that time out of clinical training impacts medical trainees’ skills, aptitude and confidence, but data is scarce [2]. The Academy of Medical Royal Colleges (AoMRC) has found that ‘whilst there is little shortage of opinion in this area, there is little clear evidence’ [3]. An observational study of 62 doctors from the United States found that the majority (67%) of doctors who had been absent from practice for 18 months or more ‘were found to have educational needs requiring moderate to considerable re-education or training ... many re-entering physicians may not be ready to jump back into practice’ [4]. In 2017, Health Education England (HEE) conducted a survey of trainees and employers via the HEE Deans, the British Medical Association the AoMRC, research bodies, the NHS Improvement and NHS England medical directors, and chief professional officers. This national survey received responses from only 53 trainees, most of whom reported a lack of confidence in their clinical knowledge and technical skills when they returned to training [1]. HEE has subsequently implemented the Supported Return to Training (SuppoRTT) initiative in recognition of the fact that targeted training and learning opportunities can help trainees return to practice in a more ‘safe and confident manner’ [1].

The abovementioned American study found that the more years the doctor was out of practice, the more likely they were to have poor performance ratings. This is particularly important for doctors taking time Out of Programme for Research (OOPR), whose breaks are often much longer than other approved mechanisms for time out of training; postgraduate research degrees typically take two to 3 years to complete. Research into the impact of returning to training after OOPR has not been disaggregated from the experiences of doctors who have taken time out of programme for other reasons, usually parental leave [1, 5].

Research into the impact of returning from OOPR is particularly relevant to integrated academic trainees. The National Institute for Health Research (NIHR) funds integrated academic training (IAT) pathways such as Academic Clinical Fellowships (ACFs) and Clinical Lectureships (CLs) to encourage the development of careers that combine research with clinical practice. Progression along the IAT pathway is dependent upon trainees obtaining a higher degree (MD or PhD), usually in an approved break from training. These trainees may continue undertaking a certain amount of clinical work whilst OOPR, which is known to reduce reports of difficulty when returning to clinical training [6]; however, this is usually capped by funders at 20% of the trainees’ time [7]. As such, trainees often cite challenges during the time out of programme and when returning to training, such as a loss of confidence in clinical skills, and lack of knowledge of changes in current practice [5].

Addressing these concerns has the potential to improve the IAT trainee experience and encourage future trainees to take time out of programme for research activities. Other stakeholders, such as patients and colleagues, will also benefit from enhanced communication, greater trainee confidence and improved knowledge and skills [1, 5].

The aims of this study were to provide early evidence of trainees’ perceptions of the challenges identified by IAT trainees around managing the return to clinical training following OOPR, and suggest potential solutions to address these issues. These findings could then be used to inform the design of larger, interventional studies with the aim of enhancing the process of returning to training after OOPR.

## Methods

Ethical approval was granted on 10th October 2018 by the University of Leeds School of Medicine Research Ethics Committee, reference MREC17–105. All methods were performed in accordance with the relevant guidelines and regulations. Informed consent to participate was obtained from all participants in the study.

### Study design

An anonymised online questionnaire was designed to establish trainees’ perceptions of the most relevant challenges of transitioning between academic and clinical training when undertaking a higher degree, and the most appropriate solutions to meet the needs of clinical academic trainees. The questionnaire was designed in a four-stage process: initial review by the research team, administration of initial draft on a sample cohort (*n* = 2), revision of questionnaire based on feedback and final external review.

The survey consisted of three parts:

- Demographics
- Potential challenges to returning to training after OOPR
- Potential solutions to the challenges of returning to training after OOPR

The latter two parts of the questionnaire were informed by the existing literature on this topic, including the HEE national survey from 2017 [1]. Potential challenges and solutions were posed to the participants, who were asked to rate whether they agreed with the proposed challenges and the extent to which the proposed interventions would be helpful. Participants were also invited to provide their own ideas and experiences of challenges and solutions in free text comment boxes. The full questionnaire is available as a Supplementary File.

### Study sample

The target sample was defined as all current IAT trainees at the University of Leeds, United kingdom, and previous IAT trainees who were currently undertaking OOPR in the local West Yorkshire region (*n* = 53). Academic Clinical Fellows were NHS employees with University of Leeds honorary contracts whilst Clinical Lecturers and most of the OOPR Clinical Fellows were employed by the University of Leeds, although some also worked for the NHS in clinical roles. The sample was not limited by clinical specialty or grade of training.

### Identification and recruitment of participants

Potential participants were identified by JB (Clinical Academic Training Programme Manager for the West Yorkshire) through the Leeds IAT programme database. Trainees were approached in two ways. Trainees who were identified as meeting the inclusion criteria for the study were sent an email invitation to participate in the survey via the Leeds IAT programme mailing list. In addition, the survey was advertised at a local academic clinicians’ conference in October 2018, where trainees were given the opportunity to complete the survey. Of the 53 trainees identified as eligible to complete the study, we aimed to approach 100% and expected a 30% response rate, in line with other questionnaire studies of this nature [8].

### Analysis of results

Demographic characteristics were summarised descriptively. Survey responses were summarised descriptively using appropriate summary statistics (frequency and percentages) and rounded to the nearest percentage point. Proportions of missing data were also presented. All analyses were carried out in SPSS (Version 23.0 or later, IBM Corp., New York, USA).

## Results

The survey was completed by 33 participants, achieving a 62% response rate. All but one of the participants were in a formal academic post (97%). The demographics of the survey population are summarised in Table 1. The proportion of participants from each specialty was similar to the specialty distribution of all the IATs.

**Table 1:** Demographics of survey participants

Gender	Male	17 (52%)
	Female	15 (46%)
	Prefer not to say	1 (3%)
Current post	Academic Clinical Fellow	12 (36%)
	Academic Clinical Lecturer	8 (24%)
	OOPR	10 (30%)
	Other^a^	3 (9%)
Stage of training	Foundation training	1 (3%)
	Core training	5 (15%)
	Specialty training ST3–5	21 (64%)
	Specialty training ST 6–8	4 (12%)
	Completed training	2 (6%)
Clinical specialty	Surgery, Anaesthesia	10 (30%)
	Medicine	8 (24%)
	Pathology	6 (18%)
	Paediatrics, Obstetrics and Gynaecology	4 (12%)
	General practice, Psychiatry, Palliative care	3 (9%)
	Dentistry	2 (6%)

^a^one participant was ‘preparing for OOPR’; one participant had returned from OOPR to a non-academic post; one participant was a part-time Clinical Research Fellow

Ten participants (30%) were OOPR at the time of survey completion. A further nine participants (27%) had previously undertaken OOPR. Of the 19 participants who were, or had been, undertaking OOPR, 15 (79%) continued to do some limited clinical work during this period. Reasons for this included: a desire to maintain clinical skills (*n* = 13); financial reasons (*n* = 8); a desire to please supervisors (*n* = 5); a desire to please clinical colleagues (n = 5); and necessity for clinical research (*n* = 3).

### Challenges of returning to training after OOPR

Participants were asked to what degree they agreed that certain challenges were relevant when OOPR and/or when returning to clinical practice. Thirty-two participants responded to this question. The proposed challenges and the survey responses are summarised in Table 2.

**Table 2:** Potential challenges of being OOPR and/or returning to training, and the extent to which participants agreed with them

Potential challenge	Degree of agreement			
	Agree	Neutral	Disagree	I don’t know
Diminishing confidence in clinical abilities and/or skills	24 (75%)	4 (13%)	1 (3%)	3 (9%)
Completing the thesis whilst transitioning back to clinical work	23 (72%)	1 (3%)	2 (6%)	6 (19%)
Rapid transition between full-time research and clinical practice	22 (69%)	5 (16%)	1 (3%)	4 (13%)
Isolation from other trainees	20 (63%)	6 (19%)	3 (9%)	3 (9%)
Becoming out of the loop regarding new clinical guidelines and protocols	20 (63%)	8 (25%)	1 (3%)	3 (9%)
Lack of available funds to update lapsed clinical courses	14 (44%)	4 (13%)	7 (22%)	7 (22%)
Reticence to ask for extra training before returning to clinical practice	14 (44%)	9 (28%)	5 (16%)	4 (13%)
Loss of communication with the Deanery/training body	10 (31%)	8 (25%)	5 (16%)	9 (28%)

Participants used the free-text option to describe other challenges which they had identified. These included: training places away from the location of continuing research; limited supervision of out-of-hours work; coping with stress and anxiety; and coping with service pressures. One trainee was concerned about ‘expectations of clinical trainers being unduly high given one may have deskilled compared to colleagues at the same training level’. This was echoed by another trainee who was ‘very worried about returning to a surgical speciality [due to] lack of operating experience’.

### Potential solutions

Participants were asked whether they believed that certain initiatives would be helpful when returning to clinical training following OOPR. Thirty-three participants responded to this question. The proposed initiatives and the survey responses are summarised in Table 3.

**Table 3:** Potential solutions to the challenges of being OOPR and/or returning to training, and the extent to which participants thought they would be helpful

Potential solution	Degree of helpfulness			
	Helpful	Neutral	Unhelpful	I don’t know
Dedicated funds allocated for the renewal of lapsed skills courses	30 (91%)	1 (3%)	1 (3%)	1 (3%)
Adequate notice (at least 12 weeks) of the next clinical rotation	30 (91%)	1 (3%)	0 (0%)	2 (6%)
Informing clinical supervisors about the trainee returning to practice	29 (88%)	1 (3%)	0 (0%)	3 (9%)
Mandatory return to standard clinical days for a period of 2 weeks	27 (82%)	3 (9%)	1 (3%)	2 (6%)
The ability for trainees to request the clinical rotation to which they return	25 (76%)	4 (12%)	1 (3%)	3 (9%)
A voluntary short-term mentorship programme pairing OOPR trainees with recent returners to practice	21 (66%)	7 (22%)	3 (9%)	1 (3%)
Priority access to study leave in the 6 months after returning to practice	20 (63%)	8 (25%)	2 (6%)	2 (6%)
Annual workshops at the regional academic conference to inform trainees about support offered	19 (58%)	12 (36%)	1 (3%)	1 (3%)
Priority access to simulation facilities and training courses locally	18 (55%)	6 (18%)	4 (12%)	5 (15%)
Mandatory Keeping In Touch (KIT) days during OOPR period	17 (52%)	7 (21%)	6 (18%)	3 (9%)
Mandatory attendance for OOPR trainees at their annual regional specialty conference	14 (42%)	8 (24%)	10 (30%)	1 (3%)
Organisation of a 6-monthly specialty-based research event	11 (33%)	11 (33%)	10 (30%)	1 (3%)

No other potential solutions were identified in the free-text boxes, although trainees were keen to highlight that support when returning to clinical practice should be personalized, and that mandatory requirements may not be appropriate: ‘I think many of the suggestions above would be helpful, but that, “mandatory” makes them difficult to support for all clinical academics, as there is such a diverse range of specialities and degree to which people continue clinical commitments during OOPR.’

## Discussion

The aims of this study were to explore the perceptions of IAT trainees regarding the challenges of the returning to clinical training following OOPR, and to suggest potential solutions to address these issues. Most of the participants agreed that the proposed challenges were relevant, especially the challenges of completing the thesis whilst transitioning back to clinical work, the rapid transition between full-time research and clinical practice, a diminished confidence in clinical abilities and/or skills and isolation from other trainees. The solutions which were thought to be most helpful included dedicated funds allocated for the renewal of lapsed skills courses, adequate notice of the clinical rotation to which trainees return, informing clinical supervisors about the OOPR trainee returning to practice and a mandatory return to standard clinical days.

This research has a number of limitations. The survey consisted of a single online questionnaire. The survey population was limited to IAT trainees from West Yorkshire, United Kingdom, in current academic roles. The results may not be applicable in other populations, for instance, OOPR trainees who are not part of an IAT pathway; however, the high levels of agreement with the proposed challenges and solutions demonstrate compatibility with the current literature base [1, 5]. The survey response rate was 62%; this response rate is high for studies of this nature, but it must be acknowledged that a proportion of the OOPR population was not represented, and there may be an element of selection bias in the results. The survey responders were anonymous, and thus it is not possible to know how many of the responders subsequently remained in academic posts. The relatively small sample size did not allow analysis of the survey responses by specialty, training level, length of time spent OOPR or whether the responders were less than full time.

## Conclusions

The survey established the most relevant challenges of transitioning between academic and clinical training when undertaking a higher degree, and potential solutions to meet the needs of clinical academic trainees. Our findings echoed those of the HEE survey which also identified diminished confidence as a challenge of returning to training [1], but has added knowledge specific to OOPR trainees such as concerns around thesis completion. This highlights the need for a personalised support programme for those returning to training, as the needs of OOPR trainees are likely to be different from those returning from parental leave, for instance. Addressing these issues has the potential to improve the trainee experience and encourage future trainees to take time out of training for research activities. Doctors, patients and other researchers will also benefit from enhanced communication, greater trainee confidence and improved knowledge and skills [5].

To this end, HEE Yorkshire and Humber has implemented a programme within the (SuppoRTT) initiative to support all clinical trainees taking time out of clinical training. There are key initiatives which address some of the issues identified by the academic trainees in this survey including:

- Access to free clinical and non-clinical return to training activities, and dedicated funding for external courses before returning to clinical practice.
- Early contact with the Trust to which the trainee will return in order to disseminate information about and oversee the return to training.
- An entitlement for trainees who have been absent for over 6 months to return to a 3 day supernumerary period.

These interventions are currently under evaluation using mixed methods studies: surveys, focus groups and interviews. Their impact on the experiences of academic trainees and the safety of patients should be addressed in future research.

## Supplementary Information

**Additional file 1: Supplementary File.** Survey questions.

## Data Availability

The datasets used and/or analysed during the current study are available from the corresponding author on reasonable request.

## Abbreviations

ACF: Academic Clinical Fellow
CL: Clinical Lecturer
HEE: Health Education England
IAT: Integrated academic training
NIHR: National Institute for Health Research
OOPR: Out of programme for research
SuppoRTT: Supported Return to Training
