# Supplementary material for: Challenges and solutions to returning to clinical training after research: a multidisciplinary survey of integrated academic trainees in West Yorkshire, United Kingdom
Source: BMC Med Educ. 2021 Feb 18;21:117. doi: 10.1186/s12909-021-02556-4 (PMC7890884; doi:10.1186/s12909-021-02556-4)
Supplement: Supplementary file 1 — Additional file 1: Supplementary File. Survey questions. [file 12909_2021_2556_MOESM1_ESM.pdf]

Challenges and solutions to returning to clinical training after research: A multidisciplinary survey of integrated academic trainees in West Yorkshire, United Kingdom

CL Downey[1], J Bentley[2], H Pandit[3]

1. Leeds Institute of Medical Research at St James's, Clinical Sciences Building, St. James's University Hospital, University of Leeds, Leeds, LS9 7TF
2. School of Medicine, University of Leeds, Leeds, LS2 9JT
3. Leeds Institute of Rheumatic and Musculoskeletal Medicine, University of Leeds, Chapel Allerton Hospital, Leeds, LS7 4SA

Supplementary File: Survey questions

p. 1 Participant Information Sheet

We would like to invite you to take part in an anonymous online survey which will take approximately **20 minutes** to complete. We would like people of all training levels and specialties to take part.

It is up to you to decide whether or not you want to take part. This information will tell you about why the project is being done and what will happen if you decide to take part. Please take as much time as you need to read and understand this information: you can talk about it with other people if you want. If there is anything you do not understand you can speak one of the project supervisors using the contact details provided.

Why is the project being done?

A number of initiatives have been launched nationally to support trainees in returning to clinical training after a period out of programme. Clinical academic trainees often have the longest periods out of programme, when they take time out of training to complete a research degree (out of programme for research, or OOPR). It is thought that this group could benefit from dedicated Return to Training opportunities to ease the transition back to clinical work. However, little is known about the specific challenges faced by clinical academics or the potential solutions to these challenges.

This survey will gather data to gain a deeper understanding of the needs of clinical academic trainees, both in training and out of programme for research (OOPR), and help to inform policy changes in the region. The survey will take approximately **20 minutes** to complete. More information on what the survey will involve is given below in the section "What will happen if I decide to take part?".

Why have I been invited to take part?

We are inviting clinical academic trainees from all levels and specialties to take part in this study. There are two groups of people that we would like to take part; if you meet the criteria for one of these groups then we would like to invite you to continue reading this information sheet.

Group 1:

Trainees who are/have been out of programme for research (OOPR) regardless of whether you have ever been employed in an integrated academic training post.

Group 2:

Trainees who are/have been employed in an integrated academic training post, e.g. Academic Clinical Fellow, Clinical Lecturer or Integrated Clinical Academic, regardless of whether you have taken time out of programme for research (OOPR).

Do I have to take part?

No, it is up to you to decide whether or not you want to take part. Before participating, you will be asked to read a consent page and give consent to take part by clicking on the ‘Next→ ’ button at the bottom of the consent page. If you start the survey but change your mind, you can withdraw at any time and without giving a reason by closing the browser window. Your responses would not be recorded. Deciding not to take part or withdrawing would not affect any support currently being offered or which might be offered to you in the future.

It is also possible for you to withdraw from the study after you have completed the survey. To do this, it is vital that you record your unique survey ID, and contact the research team with this information. You can withdraw your data anytime up to the 31st October 2018.

What will happen if I decide to take part?

If you decide to take part in this study after reading this information sheet and the consent page, this study involves completing an anonymous online survey. This can be accessed after the consent page. The survey will involve responding to a set of questions assessing your perceptions of the challenges faced by clinical academics and OOPR trainees. There is no right or wrong way of responding to the questions; simply select the response which is most true for you. The survey will include questions about different aspects of your academic life, including clinical/academic balance, challenges of OOPR and possible solutions for trainees returning to clinical practice.

What are the possible risks of taking part?

There are no known risks of taking part in this survey. You do not need to answer any questions that you do not want to. All data collected is anonymous, meaning that there is no way of identifying you from your responses.

What are the possible benefits of taking part?

We hope that the information we collect will help us find out more about the needs of clinical academic trainees, both in training and OOPR. The information collected will help to inform the development of solutions and further research projects designed to help clinical academics succeed in their professional roles.

What happens if something goes wrong?

The University of Leeds has insurance in place to cover its legal liabilities in respect of this study. If you wish to complain, or have any concerns about any aspect of the way you have been approached or treated during the course of the study, you can contact the project supervisor (contact details below). You can also contact the University of Leeds:

Clare Skinner

Head of Research Integrity and Governance

Faculty of Medicine and Health

University of Leeds

Worsley Building, Leeds, LS2 9LN

Tel: 0113 34 34897

Email: C.E.Skinner@leeds.ac.uk

Will my taking part in this project be kept confidential?

All data collected will be **anonymous**, as we do **not** ask for any personally identifying information e.g. name, address, phone number. Data will be stored securely in line with ethical and legal practices and no identifiable data will be published. All of the safeguards offered by this survey software to protect your identity (including your IP address) have been activated. Your anonymity will be preserved at all times during and after the study time period. Anonymous data may be shared with others outside of the research team for research purposes. All data will be destroyed 10 years after the study has been completed.

What will happen to the results of the research?

The results of the study will be written up by the research team for publication in health professional journals and will be presented at conferences in the UK and abroad. When we write up the results of the research, your anonymity will be preserved at all times. The results of the research will be shared with organisations who can affect change such as the University of Leeds, Heath Education England, the *Universities and Colleges Employers' Association Clinical Academic Staff Advisory Group, and NHS* Employers. The anonymised data collected from the West Yorkshire region may be shared with colleagues at the Universities of Hull and Sheffield to promote Deanery-wide improvements to integrated academic training. If this occurs, researchers will be required to obtain ethical approvals from their Institution before any access to anonymised data is granted.

Who is organising and funding the research?

The research is funded by Health Education England Yorkshire and Humber as part of a wider scheme to support clinicians returning to training after time out of programme. Ethical approval has been sought from the School of Medicine Research Ethics Committee (SoMREC/SHREC project number MREC17-105).

The research is led by Miss Candice Downey and Ms Jo Bentley. For further information, please don't hesitate to contact the investigators.

Miss Candice Downey

Clinical Research Fellow

University of Leeds

Leeds Institute of Biomedical and Clinical Sciences

Room 7.26, Level 7 Clinical Sciences Building

St James's University Hospital, Leeds, LS9 7TF

Email: c.l.downey@leeds.ac.uk

Dr Jo Bentley

Clinical Academic Training Programme Manager

University of Leeds

Room 5.27, Level 5 Clinical Sciences Building

St James's University Hospital, Leeds, LS9 7TF

Tel: 0113 206 5080.

Email: j.bentley@leeds.ac.uk

Prof Hermant Pandit

Professor of Orthopaedic Surgery (project supervisor)

University of Leeds

Leeds Institute of Rheumatic and Musculoskeletal Medicine

2nd floor, Chapel Allerton Hospital, Leeds, LS7 4SA

Tel: 0113 392 4895

Email: h.pandit@leeds.ac.uk

Thank you for taking the time to read this information sheet. Please click on the 'Next' button below to view the Consent Page and begin the survey.

p. 2 Consent form

The information on this page forms the consent form for this study. If you have any questions about the consent form, please feel free to contact the research team prior to completing the survey. By completing and returning the survey after this page, you are agreeing with the points outlined below and agreeing to take part in the study.

1. I confirm that I have read and understood the Participant Information Sheet (previous section) and have had the opportunity to have my questions answered.

2. I understand that participating in this study will involve completing an anonymous survey (starting on the next page), which will take approximately 20 minutes.

3. I understand that my participation is voluntary. I can choose not to participate or I can withdraw from the study without being penalised or disadvantaged in any way. I understand that I can withdraw from the study by simply closing the internet browser, and that if I do this, my data will not be recorded.

4. I understand that once I have submitted my responses at the end of the survey, I will be able to withdraw my data any time until 31st October 2018, and that to withdraw my data I must send my unique study ID number to one of the researchers.

5. I understand that any information I provide is confidential, and that no information that I disclose will lead to the identification of any individual in the reports on the project, either by the researcher or by any other party.

6. I understand that if quotations of mine are used in reports or publications, my anonymity will be preserved.

7. I understand that data will be stored securely, and that information will be handled in accordance with General Data Protection Regulations (2018) and the Data Protection Act (2018).

8. I understand that non-identifiable data may be shared with collaborators of the research team, for the purpose of answering additional research questions.

9. I agree to take part in the above study.

If you have any further questions about this consent form, please contact one of the project supervisors: Miss Candice Downey (c.l.downey@leeds.ac.uk) Dr Jo Bentley (j.bentley@leeds.ac.uk)

1 Do you agree to take part in the study?

- Yes
- No

p. 3 About you and your current post

2 Are you:

- Male
- Female
- Prefer not to answer

3 Are you:

- Academic Clinical Fellow
- Clinical Lecturer
- Out of Programme for Research

a If Other, please explain:

4 Which of the following have you previously undertaken? (select all that apply)

- Academic Foundation Programme
- Academic Clinical Fellowship
- Clinical Lectureship

Show all (6)

a If Other, please explain:

5 How long have you been in your current role? (months)

6 What year of clinical training are you in? If Out Of Programme for Research, what year of clinical training were you last in?

- FY1
- FY2
- CT/ST1

Show all (12)

7 What is your clinical specialty?

- Surgery/Anaesthesia/ICM
- Health Sciences (GP, Psychiatry, Palliative Med, Geriatric Med)
- Medicine (Gastroenterology, Oncology, Cardiology, Rheumatology, Rehabilitation, Endocrinology, Dermatology)

Show all (8)

a If Other, please explain:

8 Are you less than full time?

- Yes
- No

9 Do you have a formal job share arrangement?

- Yes
- No

10 Where are you/were you last mainly based for your clinical training?

- Teaching hospital
- Regional NHS Trust

- District General Hospital

Show all (4)

a If Other, please explain:

11 Where are/were you mainly based for your academic research?

- University of Leeds
- Other Higher Education Institution
- Other

a If Other, please explain:

12 What type of research are you undertaking?

- Laboratory-based
- Clinically-applied
- Medical Education

Show all (6)

a If Other, please explain:

13 Do you think that as a consequence of pursuing your academic training your clinical training will be delayed (excluding specific time taken out to do higher degree)?

- Yes
- No

a If Yes, by how long?

14 Has your academic career been impacted by the implementation of the 2016 Junior Doctors' contract?

- Yes
- No

a If Yes, please explain (if you feel comfortable doing so):

15 If you are, or have ever been, out of programme for research (OOPR), did you continue to do some clinical work?

- Yes
- No
- Not applicable

a If Yes, what motivated this? (tick all that apply)

- Financial reasons
- Desire to keep in touch with clinical skills
- Desire to please supervisors

Show all (6)

i If Other, please explain:

p. 4 Challenges when Out Of Programme for Research (OOPR) and returning to clinical training after OOPR

16 To what extent do you agree that the following challenges are relevant when Out Of Programme for Research (OOPR) and/or when returning to clinical training after OOPR?

	Agree	Neutral	Disagree	I don't know
Loss of communication with the Deanery/ training body	Checkbox	Checkbox	Checkbox	Checkbox
Isolation from other trainees	Checkbox	Checkbox	Checkbox	Checkbox
Becoming out of the loop with regards to new clinical guidelines, protocols	Checkbox	Checkbox	Checkbox	Checkbox
Diminishing confidence in clinical abilities and/or surgical skills	Checkbox	Checkbox	Checkbox	Checkbox
Lack of available funds to update lapsed clinical skills courses	Checkbox	Checkbox	Checkbox	Checkbox
Reticence to ask for extra training before returning to clinical practice	Checkbox	Checkbox	Checkbox	Checkbox
Rapid transition between full time research and returning to clinical practice	Checkbox	Checkbox	Checkbox	Checkbox
Completing the PhD or MD thesis whilst transitioning back to clinical work.	Checkbox	Checkbox	Checkbox	Checkbox

17 Can you think of any other challenges associated with returning to clinical training after OOPR?

p. 5 Potential initiatives when returning to clinical training following OOPR

18 To what extent do you think the following initiatives would be helpful when returning to clinical training following OOPR?

	Helpful	Neutral	Unhelpful	I don't know
Mandatory clinical Keeping In Touch (KIT) days during OOPR time. Activities during KIT days to be primarily decided by the trainee and work to be done on a strictly supernumerary basis. This could include arranging to 'shadow' a colleague during on-call shifts.	Checkbox	Checkbox	Checkbox	Checkbox
Mandatory attendance for OOPR trainees at their annual regional specialty conference with some focus at this event on the needs of OOPR trainees with dedicated sessions on competency requirements, clinical guidelines, etc.	Checkbox	Checkbox	Checkbox	Checkbox
Organisation of a 6-monthly specialty-based research event: an opportunity to present trainee research, meet other OOPR trainees, establish new collaborations, and discuss common issues for trainees.	Checkbox	Checkbox	Checkbox	Checkbox
A voluntary short-term mentorship programme pairing OOPR trainees with those who have completed their OOPR experience and returned to clinical training, either on an individual basis or as a network group.	Checkbox	Checkbox	Checkbox	Checkbox
Priority access to simulation facilities and local training courses at the University and local hospitals.	Checkbox	Checkbox	Checkbox	Checkbox
Priority access to Study Leave in the first 6 months of returning to training to attend courses and lectures to enhance clinical skills.	Checkbox	Checkbox	Checkbox	Checkbox
Dedicated funds allocated for the renewal of lapsed clinical skills courses, e.g. ALS (Advanced Life Support).	Checkbox	Checkbox	Checkbox	Checkbox
The ability for the trainee to request that the clinical rotation to which the OOPR trainee will return is a District General Hospital or Teaching Hospital.	Checkbox	Checkbox	Checkbox	Checkbox

Adequate notice (at least 12 weeks) of the clinical rotation to which the OOPR trainee will return, to allow maximum preparation.	Checkbox	Checkbox	Checkbox	Checkbox
Mandatory return to standard clinical days for a period of two weeks after the OOPR has ended without requirement to cover weekend and evening shifts during this time.	Checkbox	Checkbox	Checkbox	Checkbox
Inform clinical supervisors about the OOPR trainee returning to programme, and the necessity to provide enhanced support for the first two weeks after return to training.	Checkbox	Checkbox	Checkbox	Checkbox
Annual workshops at the regional Annual Academic Presentation Day (York) Away Day to inform trainees about these opportunities.	Checkbox	Checkbox	Checkbox	Checkbox

19 Are there any other initiatives which would be helpful when returning to clinical training following OOPR?

p. 9 End of survey

You have reached the end of the survey. Please click Finish.

p. 10 Final page

Thank you for your participation.
